# Proton Radiotherapy to Reduce Late Complications in Childhood Head and Neck Cancers

**DOI:** 10.14338/IJPT-20-00069.1

**Published:** 2021-06-25

**Authors:** Michael T. Spiotto, Susan L. McGovern, G. Brandon Gunn, David Grosshans, Mary Frances McAleer, Steven J. Frank, Arnold C. Paulino

**Affiliations:** Department of Radiation Oncology, University of Texas MD Anderson Cancer Center, Houston, TX, USA

**Keywords:** radiotherapy, radiotherapy, intensity-modulated, proton therapy, neoplasms, radiation-induced

## Abstract

In most childhood head and neck cancers, radiotherapy is an essential component of treatment; however, it can be associated with problematic long-term complications. Proton beam therapy is accepted as a preferred radiation modality in pediatric cancers to minimize the late radiation side effects. Given that childhood cancers are a rare and heterogeneous disease, the support for proton therapy comes from risk modeling and a limited number of cohort series. Here, we discuss the role of proton radiotherapy in pediatric head and neck cancers with a focus on reducing radiation toxicities. First, we compare the efficacy and expected toxicities in proton and photon radiotherapy for childhood cancers. Second, we review the benefit of proton radiotherapy in reducing acute and late radiation toxicities, including risks for secondary cancers, craniofacial development, vision, and cognition. Finally, we review the cost effectiveness for proton radiotherapy in pediatric head and neck cancers. This review highlights the benefits of particle radiotherapy for pediatric head and neck cancers to improve the quality of life in cancer survivors, to reduce radiation morbidities, and to maximize efficient health care use.

## Introduction

Pediatric cancers represent a unique challenge in terms of both curing the disease and minimizing long-term treatment-related complications. Radiotherapy (RT) toxicities are especially relevant to cancers involving the head and neck sites given the proximity of multiple vital organs. Because pediatric head and neck cancers are often treated with RT for improved local control because of the difficulty in achieving gross total resection, late radiation toxicities are even more relevant for this disease site because pediatric head and neck cancers represent at least 12% of all pediatric cancers and are increasing faster than pediatric cancers overall [1]. Of those patients with pediatric cancers surviving 5 years or longer, approximately 80% were treated with RT [2]. For cancers involving the head and neck area, the most common pathologic types are lymphomas, including Hodgkin lymphoma and non-Hodgkin lymphoma; neural tumors, including neuroblastoma and retinoblastoma; and soft tissue sarcomas, including rhabdomyosarcoma [1].

The types and risks of late radiation-associated toxicities are often dependent on the dose and extent of the radiation fields. Patients with leukemia, lymphoma, and neuroblastoma often receive radiation doses of less than 30 Gy and are subject to increased risk of cataracts, growth delays, dental complications, and secondary tumors. By contrast, patients with rhabdomyosarcomas, other soft tissue sarcomas, and other solid tumors, such as squamous cell carcinomas, are treated with radiation doses of 50 Gy or higher, additionally predisposing them to increased risk of hypopituitarism, bone hypoplasia, and hearing, vision, salivary, swallowing, and soft tissue toxicities.

The increased conformality and lack of exit dose in particle therapy, in particular, in proton therapy, represent an important advance to reduce the long-term radiation toxicities associated with pediatric head and neck cancers. The first part of this review discusses the advantages of proton beam therapy (PBT) over photon beam therapy in reducing late radiation toxicities, in particular, in secondary malignancies. The second part reviews the acute toxicities and long-term morbidities from PBT in pediatric head and neck cancers. Finally, we consider future applications for PBT and other modalities in pediatric head and neck cancers.

## Proton versus Photon RT for Pediatric Head and Neck Cancers

Photon beam RT has been the conventional treatment for pediatric cancers using 3-dimensional conformal radiotherapy (3D-CRT) and, recently, the more-conformal intensity-modulated radiotherapy (IMRT) or volumetric-modulated arc therapy (VMAT). Compared with 3D-CRT, IMRT had comparable locoregional control approaching ≥90% at 3 years [3–5] and fewer grade ≥3 acute toxicities in pediatric head and neck cancers. The IMRT decreases high-radiation doses to critical structures at a cost of increased integral radiation doses to a larger volume of healthy tissues.

**Figure 1.**
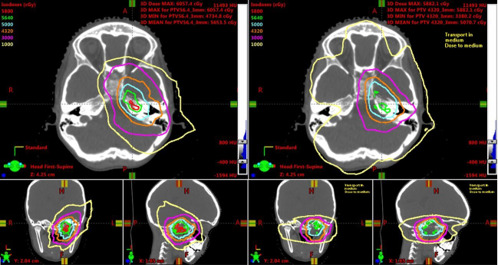
Comparison of IMPT and VMAT dose distributions. IMPT (left) and VMAT (right) plans with isodose line plans (right). Isodose lines: green: 5640 cGy; orange: 4320 cGy. Abbreviations: cGy, centigray; IMPT, intensity-modulated proton therapy; VMAT, volumetric-modulated arc therapy. Reproduced with permission from Chen et al [13].

Compared with photon-based RT, including IMRT, proton-beam RT reduces or eliminates unnecessary integral low dose radiation to surrounding structures while maintaining high conformality. Table 1 compares the published mean doses to various healthy tissues for orbital and nonorbital tumors. However, a 2012 American Society for Therapeutic Radiology and Oncology (ASTRO) consensus review [11] did not find sufficient evidence to recommend PBT outside of clinical trials in head and neck cancers and pediatric non–central nervous system malignancies. This report contrasts with the consensus of most pediatric radiation oncologists who support PBT for pediatric head and neck malignancies [12]. Furthermore, most studies forming the ASTRO consensus recommendation were based on passive-scatter proton techniques and not more-recent intensity-modulated proton therapy (IMPT) techniques, which only recently became available after those guidelines were published. Figure 1 demonstrates the dose distributions between an VMAT compared with IMPT planning.

**Table 1.** Comparison of mean radiation doses to indicated organs using IMRT or PBT for orbital and nonorbital head and neck cancers.

**Organ**	**Mean radiation dose, Gy^a^**				**Type of cancer**
	**Orbital primary**		**Nonorbital primary**		
	**IMRT**	**Protons**	**IMRT**	**Protons**	
Brainstem	NS	NS	18-26	7-8	Rhabdomyosarcoma
Optic chiasm	NS	NS	24-33	15-18	Rhabdomyosarcoma
Pituitary	15	4	33-43	24-29	Rhabdomyosarcoma
Optic nerve (ipsilateral)	37	29-45	2-37	0-30	Rhabdomyosarcoma, salivary
Optic nerve (contralateral)	NS	0	2-31	0-14	Rhabdomyosarcoma, salivary
Eye (ipsilateral)	40	25-33	1-16	1-9	Rhabdomyosarcoma, salivary
Eye (contralateral)	8	0	2-13	0-3	Rhabdomyosarcoma, salivary
Lens (ipsilateral)	32	10-44	7-9	2	Rhabdomyosarcoma
Lens (contralateral)	3	0-1	6	0-1	Rhabdomyosarcoma
Maxilla	12	7-25	30	15	Rhabdomyosarcoma
Cochlea (ipsilateral)	NS	NS	39-41	36-37	Rhabdomyosarcoma
Cochlea (contralateral)	NS	NS	29-32	4-12	Rhabdomyosarcoma
Parotid (ipsilateral)	NS	NS	38-39	31-37	Rhabdomyosarcoma
Parotid (contralateral)	NS	NS	11-24	2	Rhabdomyosarcoma

Abbreviations: IMPT, IMPT, intensity-modulated proton therapy; PBT, proton beam therapy; NS, not stated.

a Based on references 6–10.

Even with passive-scatter techniques, the volumes of low-dose RT are smaller than those of conventional photon-based RT. Leiser et al [14] demonstrated significant dosimetric sparing with passive-scatter PBT in 26 of 30 critical structures (87%) for patients with cancers in the orbital, head and neck, pelvic, and trunk and extremity sites. Similarly, a report from MD Anderson Cancer Center (Houston, Texas) compared proton and photon treatment plans for 6 children with head and neck or 18 children with brain cancers [15]. They found PBT was superior to photon RT in all cases, except for intracranial disease in which PBT demonstrated variable benefits compared with photon RT, especially for hippocampal sparing. The increasing implementation of IMPT may further increase target conformality and minimize the dose to healthy tissues. To that end, in 83 children with rhabdomyosarcoma treated with IMPT, overall nonocular grade 3 late toxicities were only 3.6%, and no grade ≥4 late toxicities were observed with PBT [14].

In head and neck rhabdomyosarcoma, PBT showed significant sparing of multiple healthy tissues, including contralateral structures such as the optic apparatus, cochlea, and both ipsilateral and contralateral parotid glands [6]. In a small case series of 7 patients with orbital rhabdomyosarcoma, PBT afforded greater local control rates, approaching 85%, with improved dosimetric sparing of the ipsilateral and contralateral optic structures and reduced optic toxicity compared with historical controls treated with photon RT [7]. That finding is consistent with other reports of favorable grade ≥3 late ocular toxicities, which have approximated 6.5% [14]. Extrapolating from adult head and neck cancers, PBT also likely reduces mucositis in pediatric head and neck cancers. Frank and colleagues [16, 17] reported delivery of 70 Gy to oropharyngeal cancers using IMPT had no grade ≥2 anterior mucositis and no grade 4–5 toxicities. A systematic review by Doyden et al [18] demonstrated that PBT better-minimized radiation doses to the salivary glands, spinal cord, brainstem, skull base structures, esophagus, and larynx compared with photon RT. Of note, the advantages of PBT persisted even when compared with the latest advances in photon treatments, including tomotherapy and VMAT [19–21]. When calculating the clinical advantage in terms of normal tissue complications probability (NTCP) in 45 cases of locally advanced head and neck cases, IMPT provided better than 10% reduction in xerostomia and mucositis in more than 50% of patients compared with IMRT or with VMAT. By contrast, a mixed photon-proton plan only reduced NTCP toxicities by approximately 10% in select cases. Tables 2 and 3 summarize the acute and late toxicities observed in children receiving photon or proton head and neck RT.

**Table 2.** Rates of acute toxicities for pediatric head and neck cancers.

**Toxicity**	**Grade ≥2 acute toxicities,^a^ %**		**Cancer types**
	**IMRT**	**Protons**	
Mucositis	91	46	Salivary
		50	Esthesioneuroblastoma
	32		Mixed head and neck tumors
		24	Sarcoma
Dermatitis	55	6.9	Salivary
		62.5	Esthesioneuroblastoma
		27	Sarcoma
Dysphagia	27	0	Salivary
		37.5	Esthesioneuroblastoma
		20	Sarcoma
Otitis externa	18	8	Salivary

Abbreviation: IMPT, intensity-modulated proton therapy.

a Based on references 9 and 22–24.

**Table 3.** Late toxicities associated with head and neck radiotherapy for pediatric cancers.^a^

**Organ and types of toxicities**	**Rates of toxicities, cumulative mean (Range), %**		**Types of toxicities**	**Reported dose when toxicity observed, Gy**
	**Photon radiotherapy**	**Proton radiotherapy**		
Dental abnormalities	35 (32-100)	7 (3-30)	Tooth, agenesis, microdontia, enamel dysplasia, xerostomia, TMJ dysfunction, osteoradionecrosis	20
Craniofacial malformations	77 (5-97)	25 (21-70)	Bone and soft tissue hypoplasia	Bone: 30 Soft tissue: 4
Hypopituitarism: GH deficiency	19 (5-40)	19 (13-22)	Decreased height, decreased bone mineralization	GH: 18
Other endocrinopathy	9 (7-9)	10 (5-10)	Delayed puberty, sexual dysfunction, subclinical hypothyroidism	GnRH: 40 ACTH: 24 TSH: 24
Optic toxicities	23 (10-83)	10 (0-14)	Cataract Keratinization Retinopathy Optic neuropathy	2 30 45 50
Hearing toxicities	19 (17-75)	7 (0-11)	High frequency hearing loss	45
Secondary cancers	3 (2-10)	1 (0-6)	Breast cancer, meningioma	1.8

Abbreviations: TMJ, temporomandibular joint; GH, growth hormone; GnRH, gonadotropin-releasing hormone agonist; ACTH, adrenocorticotropic hormone; TSH, thyroid-stimulating hormone.

a Based on references 5, 7, 14, and 25–29.

Several confounding factors may affect adequate PBT, especially IMPT, to the head and neck region. The first complex issue is the small volumes of critical organs, especially the serial structures, such as the optic nerves and chiasm, receiving a high radiation dose. Moreover, IMPT plans are usually designed either with single-field optimization or multifield optimization (MFO) in which the weighting of the spot size for all fields are optimized together. In contrast to single-field optimized IMPT, MFO-IMPT often provides more-conformal target-dose distribution and better sparing of critical structures. However, MFO-IMPT is more susceptible to both range uncertainties at the sharp dose gradient at the end of range of a proton beam as well as setup uncertainties. In the head and neck region, those uncertainties are further amplified because of intrafractional changes in soft tissue geometries, weight loss during treatment, and interfractional changes in the paranasal sinus densities. In addition, the use of radiobiological equivalent (RBE) and linear energy transfer (LET) model-based planning may better reduce possible side effect from the RBE at the end of the range [30]. To overcome the sensitivity of MFO-IMPT plans to various uncertainties, robust optimization to account for those range uncertainties is incorporated into IMPT planning [31]. Furthermore, frequent verification simulations are performed, especially for tumors adjacent to the paranasal sinuses. In addition, the differences in spot size may affect treatment planning. Smaller spot sizes may increase dose homogeneity and reduce doses to healthy tissues at the potential costs of reducing plan robustness. Consequently, a minimum monitor unit constraint is necessary to improve plan robustness. For pediatric patients, the increased proximity of critical structures and frequent cancers involving, or in proximity to, the paranasal sinuses requires extra care during treatment planning. Furthermore, improved planning may be achieved with noncoplanar beams as well as multiple beam angles. However, for tumors impinging on certain critical organs, such as the brain or brainstem, caution with PBT may be needed because the LET at the end of the range may increase the risk of symptomatic brain necrosis. Therefore, care must be taken when treating patients with PBT.

With reduced toxicities, PBT in childhood head and neck cancers has been shown to have similar outcomes as photon beam therapy. Several groups have demonstrated equivalent rates of local control for orbital and other head and neck rhabdomyosarcomas of >95% and 90%, respectively [8, 32, 33]. Although the University of Pennsylvania group reported excellent 2-year local control rates of 92.1% for photon RT and 85.4% for proton RT [22], there were no differences between proton and photon modalities on multivariate analysis. Consistent with the findings of other groups, local failure in the University of Pennsylvania cohort was associated more with both lower chemotherapy dose and lower radiation dose [32]. Similar rates of local control have been observed in other series [14, 25]. For most other head and neck cancers, efficacy of PBT has been limited to small case series [22, 33, 34, 35], and it is difficult to compare differences between modalities. Thus, extrapolating from rhabdomyosarcomas, PBT for childhood head and neck cancers provides similar efficacy as photon beam therapy.

Overall, PBT is more conformal than 3D-CRT. Furthermore, IMPT provides even improved dose distributions compared with passive-scatter PBT, and likely IMRT, as well as VMAT, especially with lower isodose lines. However, VMAT is often technically more conformal than IMPT in high-dose regions as well as in lateral dose gradients. Consequently, IMPT and VMAT planning should be compared, when possible, for optimal planning.

## The Benefit of Proton RT in Reducing Radiation-Induced Toxicities

### Acute Toxicity

Irradiation of the head and neck region is often associated with severe acute toxicities, leading to mucositis, dysphagia, and weight loss, which necessitates feeding tube placement in at least 30%–50% of cases. In a study of adults, Grant et al [9] demonstrated PBT compared with photon beam therapy reduced the rates of grade 2-3 mucositis (46% versus 91%), grade 2-3 dysphagia (0% versus 21%), and weight loss (1.2% weight gain versus 5.3% weight loss). Although rates of feeding tubes after photon RT in the pediatric population are not well established, Betchel et al [36] described 33% of pediatric patients required nutritional support after PBT. Weight loss >5% of baseline was associated with a maximum esophageal dose >50 Gy or a mean oropharyngeal dose >30 Gy. Because those patients were treated with passive scatter-beam proton RT, IMPT may potentially lower feeding tube rates. Vogel et al [22] reported on 69 children, treated with PBT to the head and neck region, predominantly with rhabdomyosarcoma or Ewing sarcoma. Grade 3 mucositis, dysphagia, and weight loss were very low at 4%, 7%, and 22%, respectively [22]. However, the benefits of PBT on mucositis, dysphagia, weight loss, and feeding tube placement require further study in the pediatric population. As a caveat, at least 1 study did not find any difference between photons and protons for acute mucosal toxicity or late mucosal toxicity, which occurred in 57% and 10% of patients, respectively [36].

Acute and late salivary toxicity is another major side effect in head and neck RT affecting young children. Acute and late salivary toxicities in children irradiated to the head and neck region are approximately 25% and 10%, respectively. Bölling et al [23] demonstrated that the dose to the submandibular gland may be important in pediatric patients because maximum doses to the submandibular gland, but not the parotid gland, were associated with acute salivary toxicity [36]. Furthermore, PBT was associated with 8.3-fold less salivary toxicity compared with photon-based RT. Similarly, for children with salivary gland tumors, PBT was associated with lower doses to the salivary glands as well as optic apparatus and the pituitary, spinal cord, mandible, oral cavity, and larynx compared with photon RT.

Although protons may reduce many of the common side effects associated with head and neck RT, one underappreciated acute side effect of PBT is increased skin toxicity; PBT is well known for the increased skin toxicity because of the challenges in controlling skin dose. This lack of skin sparing by photons over protons likely results from both the effect of additive range uncertainties for proton therapy as well as the use of more field angles and/or arc therapies for photon therapy. To that end, Phillips et al [37] reported that proton irradiation was associated with >5.7-fold risk for alopecia compared with photon irradiation. Skin sparing may be better with IMPT compared with passive-scatter proton therapy. Furthermore, the skin dose in IMPT, as in IMRT- and VMAT-based photon techniques, may be optimized by reducing target volumes several millimeters from the skin surface. However, the skin dose with IMPT is still often more robust compared with photon-based techniques, which may be even more pronounced with head and neck cancer, in which target volumes often are close to the skin.

### Late Toxicities

Given the potential for long-term survivors of childhood cancer, late toxicity must be carefully considered, both in quality and quantity of life. This concern is supported by the observation that the cumulative mortality attributable to nonrecurrence causes increases from 2% at 15 years to 7% at 30 years, whereas the mortality from recurrent cancer increases from 6.3% to 7.8% during that same time frame [38]. With a median follow-up of 10.5 years, 77% of long-term survivors of childhood head and neck cancer experience grade ≥3 late toxicities [39]. In a long-term follow-up of 17 children receiving head and neck photon RT for rhabdomyosarcoma, late effects of treatment were seen in all patients and included facial-growth retardation in 11 (65%) and dental abnormalities in 7 patients (41%) [26]. Similarly, Meazza et al [40] reported assessment of late toxicity in 36 patients treated for head and neck rhabdomyosarcoma with photon-based RT. The most common side effects were facial growth retardation (72%; 26 of 36) and dental abnormalities (69%; 25 of 36). Xerostomia occurred in 38% of all patients [40]. Furthermore, visual or orbital toxicities occurred in 3 of 11 (27%) 5-year survivors of nonorbital head-and-neck rhabdomyosarcoma treated with photon RT [26]. In addition, in a similar patient population, auditory toxicities were observed in 20% of patients treated with photon RT.

By contrast, PBT appears to substantially reduce late radiation complications because grade ≥2 and ≥3 late toxicities were 35% and 17% at 10 years and 45% and 17% at 20 years, respectively [41]. Fukushima et al [27] followed 60 patients 15 years or younger treated between 1983 to 2011 with PBT. A total of 32% (19 of 60) of patients had ≥1 grade ≥3 toxicity, most commonly associated with facial deformities and/or central nervous system damage. By contrast, the severity of other late toxicities, including hormone deficiencies, hair loss, hypothyroidism, and dental dysgenesis were mostly grade 1-2. Similarly, Leiser et al [14] reported low rates of grade 3 toxicities at 5 years for ocular and nonocular rhabdomyosarcoma of 18.4% and 3.6%, respectively.

Hearing loss represents another significant morbidity for children irradiated for head and neck cancers. The combined Intergroup Rhabdomyosarcoma Studies (IRS) II and III reported a 17% rate of hearing loss [28]. Neuro-otologic morbidity is, in part, related to cochlear irradiation with maximum radiation doses kept to <32 Gy to minimize the risk of hearing loss [42]. Extrapolating from medulloblastoma, IMRT reduced cochlear doses compared with conventional RT among 26 children treated for medulloblastoma [43]. However, there were no differences in hearing loss between photons and protons likely because of the inclusion of cisplatin and/or other ototoxic chemotherapies [29, 44]. By contrast, Moeller et al [45] reported a very low rate of ototoxicity in children with medulloblastoma treated with PBT. These results must be interpreted cautiously, however, because of the role of ototoxic chemotherapy in medulloblastoma management.

### Radiation-Induced Malignancies

Radiation-associated malignancies are one of the most feared complications in irradiating pediatric cancer patients. For survivors of childhood cancers, the 20- and 30-year risk for developing second malignancies approximates 3.2% and 7.9%, respectively [46, 47]. The risk of second malignancies continues to increase, even after the age of 50 years, with a cumulative incidence of 16.3% by age 55 years [48]. However, PBT has consistently decreased the estimated radiation-induced cancer risk in pediatric patients. Leiser et al [14] observed only 1 radiation-induced malignancy in 83 patients (1%) with rhabdomyosarcoma. Reporting the 10-year cumulative incidences of secondary tumors is survivors of retinoblastoma, PBT was associated with significantly lower in-field malignancies (0% versus 14%) and all malignancies (5% versus 14%) compared with photon RT [49]. Mizumoto et al [41] reported long-term follow-up for 62 patients treated with PBT followed for more than 5 years; of which, 46 (74%) were treated to the head and neck and brain regions. No secondary tumors occurred within the irradiated field.

Because secondary malignancies are difficult to assess in longitudinal studies, given the relative recent widespread implementation of proton therapy, much effort has been devoted to estimating metrics for cancer risk based on dosimetric assessment of RT plans. However, Ngyuen et al [50] has questioned the reliability of those cancer risk models because of high degrees of uncertainty in dosimetry. Even though those uncertainties limit the validity of cancer risk models for a single modality, comparisons of cancer risks between proton and photon treatment plans can still be made because the ratio of absolute risks between 2 modalities is less sensitive to those uncertainties. In a prospective, phase II study comparing the integral dose for passive-scatter PBT versus IMRT for rhabdomyosarcoma in 54 patients, the integral dose for IMRT was 1.8 times greater in the head and neck region and 3.5-fold greater for orbital site [10, 51]. In addition, IMPT in parameningeal head and neck rhabdomyosarcoma reduced the estimated risk of secondary cancers by 1.75-fold compared with passive-scattered protons, 2-fold compared with IMRT, and 2.5-fold compared with 3D-CRT [52]. Similarly, compared with photon plans, Stokkevåg et al [53] demonstrated that IMPT achieved significantly better dose conformity, resulting in a 6-fold reduction in risk of second malignancies.

Moteabbed et al [54] calculated the lifetime attributable risk (LAR) of second cancers in pediatric patients irradiated with passive-scatter PBT, IMPT, IMRT, or VMAT. The LAR for soft tissue or skull malignancies ranged from 0.01% to 2.8% for PBT and 0.04% to 4.9% for photon-based therapy [54]. Of note, that LAR was independent of the number of fields used for proton or photon RT. Although the authors did not find a difference in cancer risk between passive-scatter PBT or IMPT, their study calculated the lifetime risk for proton modalities using a 12-mm spot size. That larger spot size may not have fully realized the advantages of IMPT delivered with smaller spot sizes. To that end, Moteabbed et al [55] demonstrated that IMPT using larger spot sizes did not provide a dosimetric advantage over passive-scattered proton beams. Decreasing the spot size lowered the mean doses to healthy tissues by up to 11.6%, providing a maximum NTCP reduction of 5.4% relative to passively scattered PBT [55].

Not all reports have demonstrated reduced risks of secondary cancer with PBT. Tamura et al [56] calculated the LAR for PBT and IMRT for 4 anatomic sites, including the head and neck region. For the brain, head, and neck region, the difference in lifetime risk between PBT and IMRT was 1.02% ± 0.52%. Many factors may have caused the discrepancies between this report and other series. First, of the 8 cases used to estimate the LAR for irradiation of the head and neck region, 7 cases (88%) were primary brain tumors, which are associated with similar LARs between photon and proton modalities. One case was a patient with Ewing sarcoma involving the head and neck. Second, the LAR was estimated for passive-scatter PBT only and may have missed a greater benefit with IMPT. Thus, most of the evidence indicates that PBT reduces integral dose and thereby likely reduces the risk of secondary malignancies.

### Children with Genetic Conditions That Sensitize Them to Radiation Side Effects

Pediatric head and neck malignancies are rare, with an annual rate of approximating 1 in 100 000 person-years [1]. Although genetic diseases associated with DNA repair and the maintenance of genomic stability are also rare, cancers including those involving the head and neck region frequently arise in pediatric and young adult patients. One of the most well-known genetic diseases is Fanconi anemia, which occurs from defects in a cluster of ≥17 genes involved in homologous recombination and occurs with an incidence of 1 in 130 000 births. Adolescents and young adults with Fanconi anemia frequently develop head and neck squamous cell carcinomas, with an incidence of up to 50% by 40 years old [57]. Because of defects in DNA repair, patients with Fanconi anemia and head and neck squamous cell carcinoma tolerate chemotherapy and radiation poorly, even though they frequently present with locally advanced disease [58]. Similarly, Li-Fraumeni cancer syndrome, a germline disorder in p53, has been associated with many pediatric head and neck cancers, including rhabdomyosarcomas [59], soft tissue sarcomas [60], and squamous cell carcinomas. Clinical and preclinical evidence suggest that Li-Fraumeni syndrome may be associated with increased treatment toxicities, including secondary malignancies [61].

Consequently, PBT may benefit children with cancers resulting from germline disorders by increasing conformality and reducing integral dose. At this point, only case reports support the feasibility of PBT in these settings. Hartman and Hill-Kayser [62] describe the experience using proton therapy to treat an oropharyngeal squamous cell carcinoma in a child with dyskeratosis congenita, which is frequently caused by a mutation in the telomere-associated protein DKC1. Although the patient still experienced greater dermatitis and mucositis, the patient remained disease free and was no longer dependent on a feeding tube. Similarly, PBT successfully controlled a recurrence of a choroid plexus tumor in a 3-year-old child with Li-Fraumeni syndrome [63]. Finally, Beckham et al [64] described the first Fanconi anemia patient treated with proton RT for head and neck cancer who tolerated the treatment remarkably well. Although PBT may benefit patients with cancers associated with genetic syndromes, care must be taken when determining radiation dose with PBT. Namely, disruption of DNA repair pathways, especially those involving homologous recombination, may alter the relative biologic effectiveness of particle therapy beams [65, 66]. Consequently, further study is necessary to define the advantages of proton therapy in children with germline mutations.

### Socioeconomic Implications of PBT in Children

Although proton therapy can be implemented for many different types of cancer, PBT requires greater health care expenditures and may not be accessible to all individuals at this time. Dvorak et al (unpublished data) demonstrated that, for a single institution, treating patients with PBT instead of IMRT or VMAT increased costs by 22%. However, in the entire patient population, head and neck pediatric cancers represented only 16% and 2% of the entire population treated with proton or photon radiation, respectively. Verma et al [67] demonstrated the cost effectiveness of PBT for both pediatric patients as well as high-risk head and neck cancers. However, disparities exist for access to PBT for children, which depend, in part, upon insurance, race, household income, and/or parental educational level [68, 69]. Consequently, the benefit of proton therapy for children is likely tempered by issues with access-appropriate treatment facilities.

Despite the theoretic and empiric advantages of PBT over photon-based therapy, it remains unclear what the ethical limitations are for ensuring pediatric head and neck cancers are treated with PBT. Johnstone et al [70] argued that the dosimetry, as well as emerging clinical evidence, argues that PBT is the only ethical approach for craniospinal irradiation of children. By contrast, Wolden [71] argues that the existing data should be balanced with the burden of relocation for treatment. Consequently, the downstream costs of treating secondary cancers, craniofacial abnormalities, and other late complications, including dental hypoplasia, likely outweigh the upfront financial toxicity of PBT.

### Alternative Particle-Based Modalities

Moreover, PBT remains the “gold standard” for particle-based therapies that minimize toxicities with equivalent efficacy as photon RT in children with cancer. The recent emergence of IMPT may further improve proton beam delivery for head and neck cancers. The advancement of MFO-IMPT and the potential for LET optimization may hold additional promise to provide greater conformality, reduce heterogeneity, and further reduce toxicities as well as secondary malignancies.

**Figure 2.**
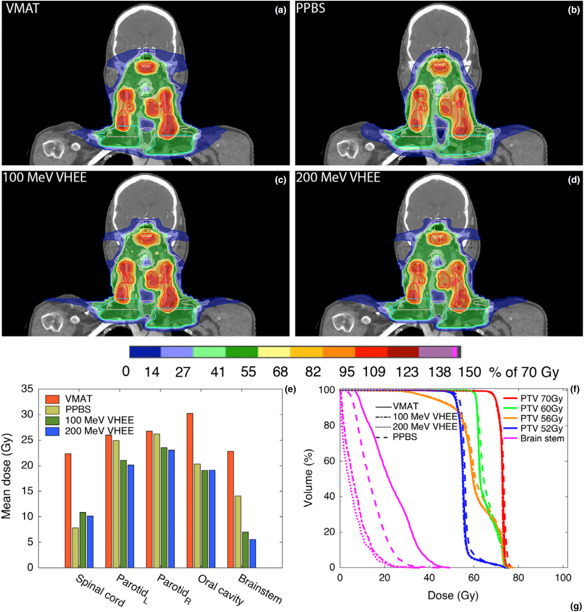
Comparison of VMAT, PPBS, and VHEE planning. (a–d) Coronal images through PTV for the different modalities: (a) VMAT, (b) PPBS, (c) 100 MeV VHEE and (d) 200 MeV VHEE. (e) mean doses to the spinal cord, parotid glands, oral cavity, and brain stem, (f) dose volume histogram for the PTVs and brain stem. Abbreviations: PPBS, proton pencil-beam scanning; PTV, planning target volume; VHEE, very high energy electron; VMAT, volumetric-modulated arc therapy. Reproduced with permission from Schuler et al [74].

Carbon ion therapy has demonstrated impressive control rates in a small series of children and adolescents with rhabdomyosarcomas [72], as well as in chordomas and chondrosarcomas [73]. However, the few centers offering carbon ion therapy worldwide limits current research into this modality in pediatric head and neck cancers. In addition, the dosimetry of very high energy electrons (VHEEs), with energy ranging from 100 to 200 MeV, has been shown to be superior to the dosimetry of VMAT. Figure 2 demonstrates potential benefits of VHEE dosimetry compared with proton and photon RT. Furthermore, VHEEs have demonstrated better theoretic sparing of central nervous structures compared with pencil-beam proton therapy [75]. Although both carbon ion therapy and VHEE require much further study, both modalities present interesting theoretic benefits to further reduce the toxicities associated with pediatric head and neck RT.

## Conclusions

As long-term survival rates of children with cancer increase, so do the children at risk for long-term radiation morbidities. Irradiation of the head and neck region is associated with multiple radiation complications afflicting the vision, hearing, eating, and growth. It remains unclear the extent to which particle beam therapy, especially proton therapy, represents a cost-effective approach to minimize several dosimetric measures of toxicity that have been realized in the clinic. However, PBT has demonstrated few acute and late radiation toxicities and provides similar rates of locoregional control for pediatric patients with head and neck cancer. In addition, PBT may benefit children with genetic syndromes that both sensitize them to radiation side effects and predispose them to head and neck cancers. Emerging technologies provide theoretic benefit for further reducing radiation toxicities. Thus, improving the technical precision of radiation and medical management of radiation toxicities gives hope to the survivors of all pediatric cancers, including those of head and neck, who are at risk for, or are suffering from, the complications of RT.
